# Cor Triatriatum Sinister diagnosed in adult life with three dimensional transesophageal echocardiography

**DOI:** 10.1186/1471-2261-10-54

**Published:** 2010-10-28

**Authors:** Righab Hamdan, Nicolas Mirochnik, David Celermajer, Pierre Nassar, Laurence Iserin

**Affiliations:** 1 Saint Antoine Hospital, Paris, France; 2 European hospital Georges Pompidou, Paris, France; 3 Scandrett Professor of Cardiology, University of Sydney Clinical Academic Cardiologist, Royal Prince Alfred Hospital, Sydney

## Abstract

**Background:**

Cor triatriatum is a very rare congenital abnormality, usually symptomatic during childhood, diagnosis in adult age is less common.

**Case Presentation:**

We report the case of a 40 years old woman referred to our hospital for atrial flutter ablation, transthoracic cardiac bidimensional echocardiography showed an abnormal membrane bisecting the left atrium, the diagnosis of cor triatriatum was fully made via three dimensional transesophageal echocardiography. More interstingly three other cardiac anomalies were associated: ostium secundum atrial septal defect, dilated coronary sinus due probably to persistent left superior vena cava and normally functioning bicuspid aortic valve.

**Conclusions:**

Cor triatriatum sinister in adult life is important to recognize because it may be easily surgically correctable when hemodynamically significant. Three Dimensional transesophageal echocardiography is a minimally invasive and highly sensitive diagnostic modality.

## Background

Cor triatriatum is among the rarest of all congenital cardiac anomalies accounting for 0.1-0.4% of congenital heart disease [1,2]. In this malformation the left atrium is divided by an abnormal fibromuscular diaphragm into a posterosuperior chamber or embryonic common pulmonary vein, receiving the pulmonary veins and an anteroinferior chamber or embryonic left atrium giving rise to the left atrial appendage and leading to the mitral orifice [3]. The two chambers generally communicate through one or more openings in the intra-atrial membrane [1,2]. This lesion is usually symptomatic during childhood (symptoms of left heart obstruction or arrhythmia). A minority of individuals present in adulthood when the diagnosis is made incidentally.

We report the case of a 40 years old woman hospitalized for atrial flutter ablation and in whom cor triatriatum was incidentally diagnosed.

## Case presentation

A 40 years old woman was referred to our hospital for atrial flutter ablation. In her cardiac history we noted uncomplicated repair of an ostium secundum atrial septal defect at the age of 5 years. In the last year, she had had many episodes of paroxysmal palpitations and in the last four months she had also experienced worsening dyspnea and persistent palpitations. Atrial flutter was diagnosed and persisted despite antiarrhythmic therapy (Amiodarone and Beta-blockers). She was then referred to our hospital for flutter ablation

**Figure 1:**
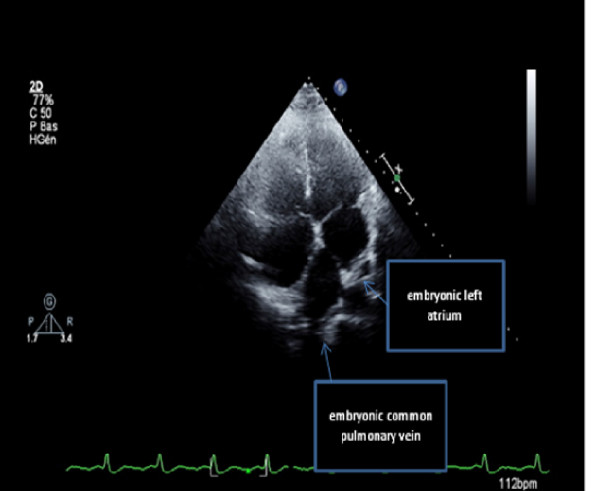
**Two D TTE showing the membrane bisecting the left atria**.

**Figure 2:**
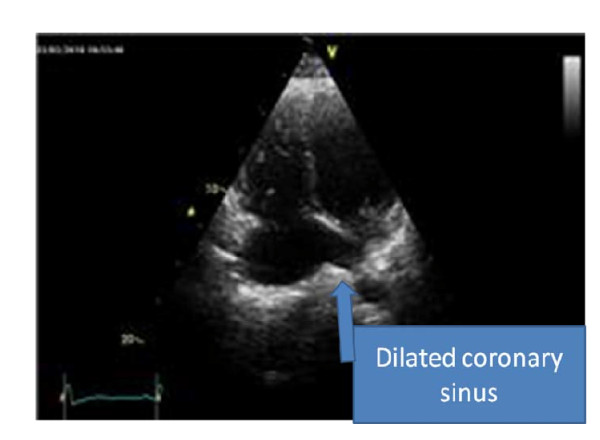
**Dilated coronary sinus due probably to PLSVC was easily seen on 2 D TTE**.

**Figure 3:**
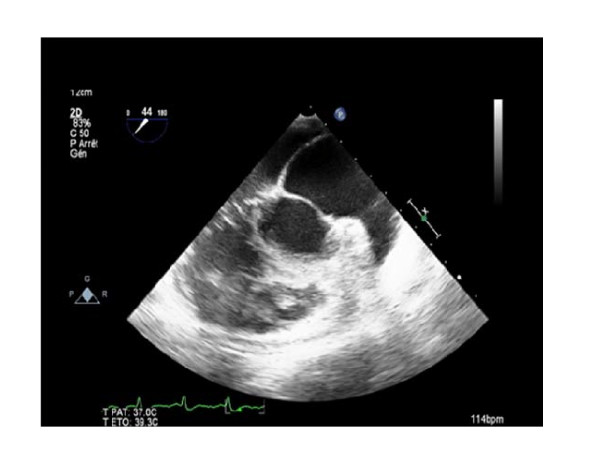
**Three D TEE clearly showing the membrane with the anteroinferior chamber, the left atrial appendage and the mitral orifice**.

**Figure 4:**
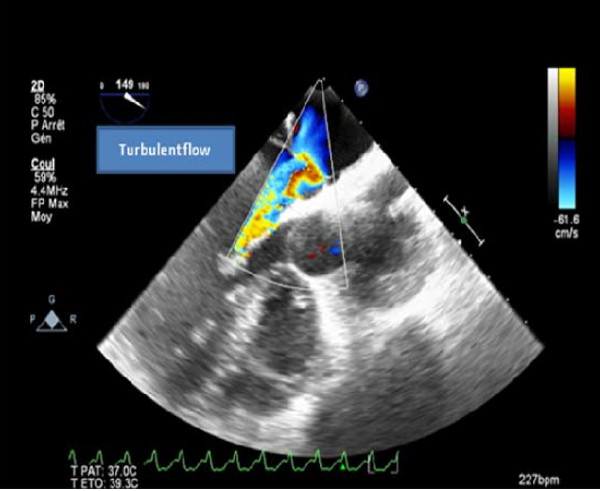
**Color flow, 3 D TEE showing turbulent flow across the orifice**.

**Figure 5:**
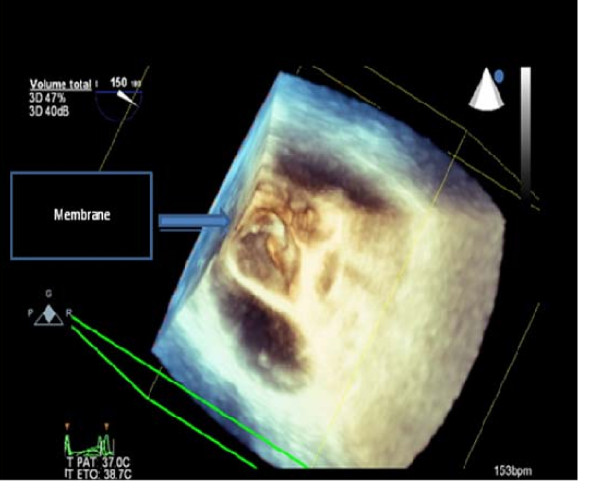
**Crescent shape of the membrane view from the pulmonary veins on 3 D TEE**.

**Figure 6:**
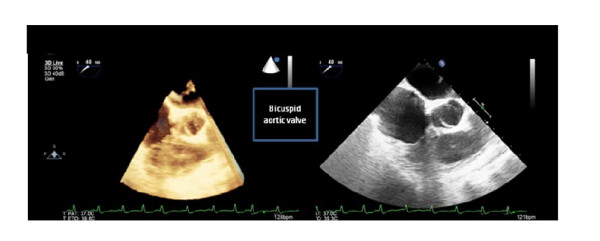
**icuspid normally functioning aortic valve seen in our patient is an unusual finding**.

On physical examination she had regular rapid pulse at 120 beat/min and normal blood pressure. Cardiac auscultation was normal. The EKG showed atypical atrial flutter with variable atrioventricular conduction (2/1 or 3/1). Blood test results were unremarkable. Transthoracic echocardiography (TTE) showed an abnormal membrane bisecting the left atrium into 2 chambers (Figure 1) and a dilated coronary sinus because of persistent left superior vena cava left (PLSVC) (Figure 2). Two dimensional (2D) transesophageal echocardiography (TEE) (Figure 3) clearly showed the membrane dividing the left atria into two chambers, with the left atrial appendage. Color Doppler showed turbulent flow (Figure 4) and three dimensional (3D) TEE (Figure 5) showed the crescent shape of the membrane, while the continuous wave Doppler across the membrane showed a diastolic intra atrial mean gradient of 8 mmHg. Our patient also had a normally functioning bicuspid aortic valve (Figure 6).

A successful radiofrequency flutter ablation (cavo-tricpuspid isthmus ablation) was performed and the patient was discharged in sinus rhythm with oral anticoagulant and beta blockers.

## Discussion

We reported a unique case of cor triatriatum associated bicuspid aortic valve, ostium secundum atrial septal defect and dilated coronary sinus probably due to PLSVC. To the best of our knowledge, this is the first case describing all four lesions in the same patient.

Cor triatriatum was first described by Church in 1868 [4], the membrane of cor triatriatum appears as a linear echo bisecting the left atrium. Several classification schemes have been proposed for describing cor triatriatum; the simplest was proposed by Loefller in 1949 [5]. It is based on the number and size of fenestrations in the fibromuscular membrane dividing the left atrium: group 1 is defined as having no opening, group 2 as having one or more small openings and group 3 as having a single, large opening.

The embryologic etiology remains debated, it may result from incomplete incorporation of the common pulmonary vein into the left atrium [1,4,6], abnormal overgrowth of septum primum[1,4], entrapment of the common pulmonary vein by the left horn of the sinus venosus preventing its incorporation into the left atrium [1], or persistence of a left-sided superior vena cava which may impinge on the left atrium, resulting in the formation of an abnormal membrane [4].

Most frequent cardiovascular abnormalities associated with Cor triatriatum sinister in adult are usually related to the spectrum of left heart obstruction [5]: mitral regurgitation and supravalvular annulus [4,5], and PLSVC draining in the coronary sinus [5,7]. Other abnormalities are ostium secundum atrial septal defect, and less commonly aortic regurgitation with dissecting aneurysm (bicuspid aortic valve?) [5] and anomalous partial pulmonary venous return [4,5,8].

Although cor triatriatum is part of the spectrum of left heart obstruction there are very few reports in literature about an association of cor triatriatum with aortic bicuspid valve [8,9], thus our case is a unique association of four abnormalities, three of them being mildly symptomatic up to adult age. Symptoms appeared, as in moderate mitral stenosis, during sustained atrial arrhythmia.

The diagnosis is usually established by 2 D TTE. Color flow mapping usually demonstrates increases in velocity and turbulent flow, suggesting obstruction that can be assessed by continuous wave Doppler through the membrane [4,10]. Transesophageal echocardiography is superior to transthoracic imaging to diagnose cor triatriatum, providing better imaging of the left atria, left atria appendage, morphology of the dividing membrane and the degree of obstruction [6]. Three D echocardiography is a more recent diagnostic tool providing additional information, able to demonstrate the entire membrane, the size, the location and the number of openings in the dividing membrane [6,10]. In our patient 2 D TTE was abnormal but could not fully assess the diagnosis, which was finally confirmed on TEE3 D echocardiography. Associated lesion (bicuspid aortic valve) was also assessed by this technique.

Surgical resection of the intraatrial membrane is indicated with severe obstruction as for severe mitral stenosis [1,6]. Successful percutaneous balloon dilation has been described but surgery remains the gold standard in the management of symptomatic cor triatriatum sinister [1].

## Conclusion

Although rare, Cor triatriatum sinister in adult life is important to recognize because it may be easily surgically correctable when hemodynamically significant. Three D TEE is a recent, minimally invasive and highly sensitive diagnostic modality.

## Competing interests

The authors declare that they have no competing interests.

## Authors' contributions

RH, and LI contributed in the literature review, writing and correcting the manuscript, and echographic images acquisition. NM helped in echographic images acquisition and interpretation, manuscript correction. PN helped in the literature review, manuscript writing and correcting. DC helped in the manuscript writing and correcting. All authors read and approved the final manuscript.

## Pre-publication history

The pre-publication history for this paper can be accessed here:

http://www.biomedcentral.com/1471-2261/10/54/prepub
